# Percutaneous Atrial Septostomy in Adult Patients on Veno-Arterial Extracorporeal Membrane Oxygenation for Cardiogenic Shock: A Canadian Single-Center Experience

**DOI:** 10.3390/jcm13237433

**Published:** 2024-12-06

**Authors:** Nidal El Yamani, Siddhartha Mengi, Mario Sénéchal, Eric Charbonneau, Maxime Laflamme, Julio Farjat-Pasos, Josep Rodés-Cabau, Jean-Michel Paradis

**Affiliations:** Quebec Heart and Lung Institute, Laval University, Quebec City, QC G1V 4G5, Canada; nidal.el.yamani@umontreal.ca (N.E.Y.); siddhartha.mengi.1@ulaval.ca (S.M.); mario.senechal@criucpq.ulaval.ca (M.S.); eric.charbonneau@criucpq.ulaval.ca (E.C.); maxime.laflamme@criucpq.ulaval.ca (M.L.); julio-ivan.farjat-pasos.1@ulaval.ca (J.F.-P.); josep.rodes@criucpq.ulaval.ca (J.R.-C.)

**Keywords:** cardiogenic shock, left ventricular unloading, balloon atrial septostomy, pulmonary edema, veno-arterial extracorporeal membrane oxygenation

## Abstract

**Background/Objectives:** Patients with cardiogenic shock on veno-arterial extracorporeal membrane oxygenation (VA-ECMO) frequently develop left ventricular (LV) distension and pulmonary edema due to an increased LV afterload. A balloon atrial septostomy (BAS) is a technique used to alleviate LV pressure and facilitate left atrial decompression. While primarily performed in pediatric populations, this procedure’s feasibility in adult patients is less studied. This study aimed to evaluate the procedural outcomes, including the safety and effectiveness, of BASs in adult patients with cardiogenic shock supported by VA-ECMO. **Methods:** This single-center retrospective study included 11 adult patients with cardiogenic shock on VA-ECMO, who underwent a BAS between 2012 and 2023. Multiple parameters were used to evaluate the global clinical impact of a BAS on patients with cardiogenic shock. **Results:** Between 2012 and 2023, 11 patients with cardiogenic shock on VA-ECMO underwent a BAS procedure in our institution. The mean time from the BAS to advanced therapy was 6.4 days. Procedural success was achieved in all patients with no complications. Nine patients (82%) had an improvement in PaO_2_/FiO_2_ 24 h post-BAS procedure. All patients had an improvement in the pulmonary edema on the chest X-ray 24 to 48 h after the procedure, with clear radiography achieved in nine patients (82%) in a mean time of 7 days (range: 1.5–13 days). A total of five patients (45%) had in-hospital mortality due to non-procedural complications and the mortality timing from BAS was between 5 to 23 days. Among those discharged, all six patients were alive at the 1-year follow-up. **Conclusions:** A BAS is a feasible and safe technique for decompressing the left atrium in adult patients on VA-ECMO. It significantly improved pulmonary edema and oxygenation in most cases. Further studies with larger populations are needed to evaluate its impact on long-term outcomes.

## 1. Introduction

Veno-arterial extracorporeal membrane oxygenation (VA-ECMO) provides mechanical circulatory support for patients with refractory cardiogenic shock, facilitating myocardial recovery and supporting end-organ perfusion [1]. However, the arterial outflow from the ECMO circuit can increase the left ventricular (LV) afterload, leading to an increase in the LV end-diastolic and left atrial (LA) pressures that can result in pulmonary edema. Also, the rising pressures increase the wall stress and myocardial oxygen consumption, thereby increasing the risk of myocardial ischemia [2]. Available data show that up to 60% of patients on VA-ECMO develop severe LV distension, with the highest proportions in neonates and children [3].

The benefit of LV decompression is based on two potential mechanisms: (1) reducing the LA pressure, which promotes early lung recovery and shortens the duration under sedation and ventilation, and (2) improving the LV wall stress, which increases subendocardial perfusion, lowers myocardial oxygen consumption, and facilitates LV functional recovery [4]. LA venting strategies, such as a balloon atrial septostomy (BAS) and trans-septal cannula, have been mostly described in the pediatric population, demonstrating significant improvements in LV/LA hemodynamics and clinical outcomes [5]. However, only a few studies have demonstrated the feasibility of BAS in the adult population. The main objective of our study was to report our single Canadian center experience with percutaneous BASs in adult patients on VA-ECMO for cardiogenic shock.

## 2. Methods

### 2.1. Study Design and Population

A retrospective chart review was performed on all patients with cardiogenic shock who received VA-ECMO between 2012 and 2023 at the Quebec Heart and Lung Institute, Quebec, Canada. From this group, patients who underwent a percutaneous BAS were selected, which resulted in a cohort of 11 patients. Patients were selected for a BAS based on the presence of refractory pulmonary edema and significant LV overload despite maximal pharmacologic and mechanical support on VA-ECMO. A BAS was considered an appropriate decompression strategy for cases where excessive LV afterload was contributing to worsening pulmonary congestion and posed a high risk to patient stability. We excluded (1) patients on ECMO for reasons other than cardiogenic shock and (2) patients on micro-axial pumps (e.g., Impella [Abiomed, Danvers, MA, USA]), LV/LA cannula (inserted percutaneously or surgically), or any other LV venting strategies.

### 2.2. Study Endpoints

The aims of this study were (1) to describe the technical/procedural considerations of a BAS in adults and (2) to evaluate the global clinical impact of a BAS on patients with cardiogenic shock on VA-ECMO, by examining different parameters: peri-procedural complications, the evolution of pulmonary edema on a chest X-ray, the ratio of the partial pressure of oxygen in arterial blood (PaO_2_) to the fraction of inspiratory oxygen concentration (FiO_2_), ECMO flow, in-hospital length of stay, intensive care unit (ICU) length of stay, in-hospital mortality, 30-day mortality, 1-year mortality, cardiac function recovery (defined as LV ejection fraction [LVEF] > 40%), and the use of advanced therapies (left-ventricular assist device [LVAD], heart transplant).

### 2.3. Technique Description

All procedures were performed in the catheterization laboratory or the hybrid operating room under fluoroscopic and transesophageal echocardiography (TEE) guidance. A standard trans-septal puncture technique was used. Usually, the right femoral vein was punctured. A SL1 or a VersaCross catheter was then positioned in the superior vena cava and brought back into the fossa ovalis of the interatrial septum. The trans-septal puncture was performed using a radiofrequency needle loaded in the sheath or the VersaCross dedicated wire. Then, a 0.035-inch stiff guide wire replaced the needle and was advanced into the left upper pulmonary vein. After withdrawal of the sheath, a 10–16 mm balloon was then advanced until its mid-portion was positioned across the atrial septum and inflated with diluted contrast. To minimize the risk of tearing the interatrial septum and achieving a non-restrictive atrial septal defect (ASD), the balloon size was tailored to each patient based on the individual anatomical and clinical factors, such as the interatrial septal thickness, the degree of LA decompression required, and the patient’s body habitus. Larger balloons were selected for patients with more significant interatrial septal resistance to achieve effective decompression, while smaller balloons were used in patients with thinner septal walls to minimize the risk of over-dilation. Under TEE guidance, the center of the balloon was positioned on the interatrial septum and then inflated, which created an ASD with the left-to-right shunt visualized by color-flow Doppler. Additional balloon inflation (usually with a bigger balloon) was performed if the left-to-right shunt was not deemed to be sufficient by the echocardiographer. Procedural success was defined as the successful creation of an ASD and the visualization of continuous and unrestrictive left-to-right shunting on color-flow Doppler imaging. The procedural time was calculated from the puncture of the right femoral vein to the withdrawal of the guide wire. Potential procedural complications, such as cardiac perforation, atrial septal tear, atrial appendage tear, cardiac tamponade, valvular injury, vascular injury, arrhythmia, and embolic events, were reported. After the equipment was removed, the venous access site was closed with a figure-of-eight skin suture or manual pressure.

## 3. Results

Between 2012 and 2023, 11 patients with cardiogenic shock on VA-ECMO underwent BAS at our institution. A total of 72% were male, and the mean age was 54 ± 10 years. In most patients, the underlying etiology of cardiogenic shock was acute coronary syndrome. Other causes included arrhythmia, myocarditis, and postpartum cardiomyopathy (Table 1).

The mean time from ECMO initiation to the BAS procedure was 2 days (range: 0–6 days), and the average procedure duration was 51 min (range: 30–70 min). Procedural success was achieved in all patients without complications (Table 1). Patient #4 underwent an initial BAS procedure with an ASD of 10 mm, but the result was not durable, as it closed 3 days later, resulting in worsening pulmonary edema. He had a successful redo BAS with an ASD of 15 mm.

**Figure 1:**
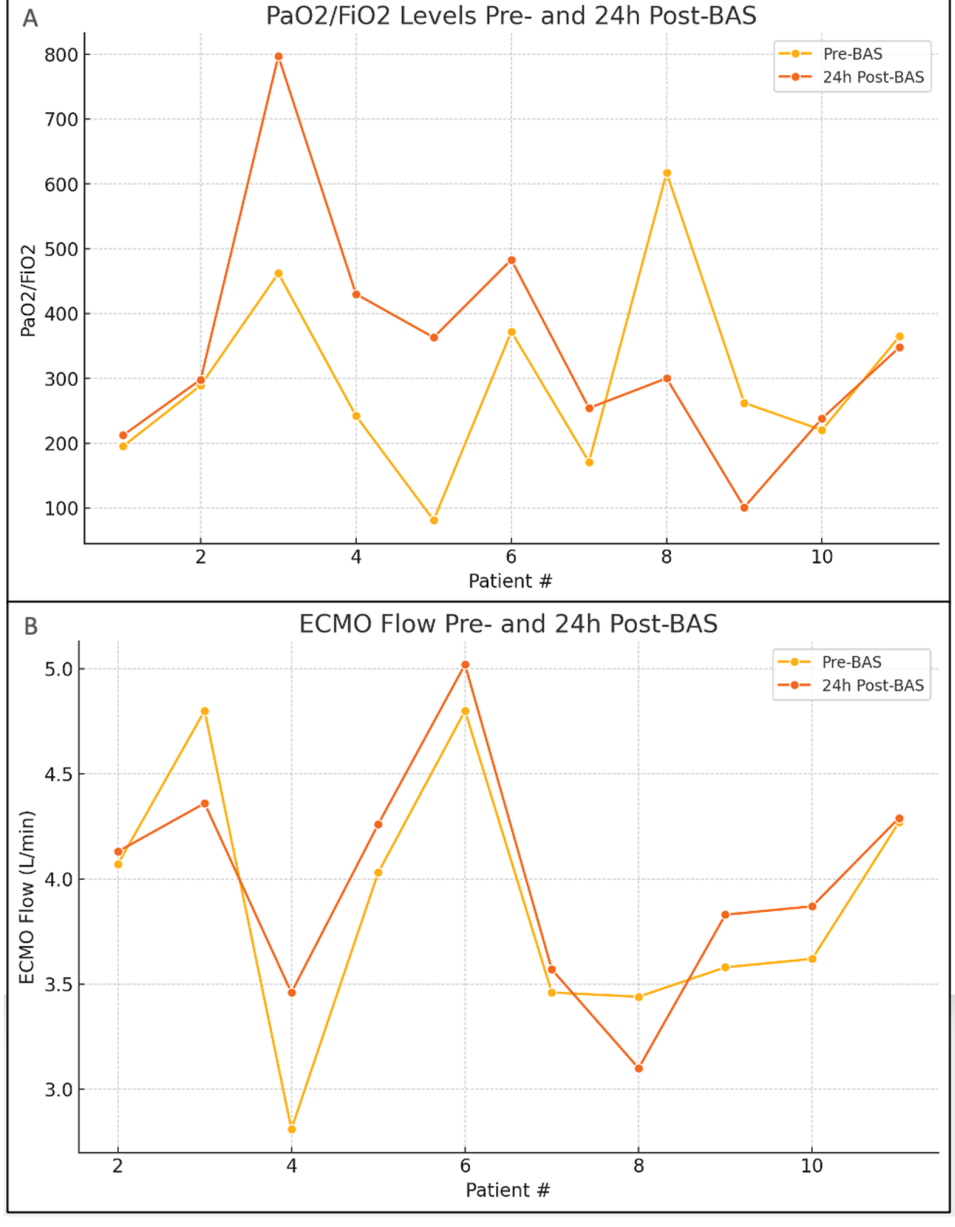
Pre- and post-balloon atrial septostomy (BAS) clinical parameters in adults on veno-arterial extracorporeal membrane oxygenation (VA-ECMO) for cardiogenic shock. (**A**) PaO_2_/FiO_2_ levels pre- and 24 h post-BAS: the top graph shows the PaO_2_/FiO_2_ levels for each patient before and 24 h after the BAS procedure. (**B**) Extracorporeal membrane oxygenation (ECMO) flow pre- and 24 h post-BAS: the bottom graph illustrates the ECMO flow rates before and 24 h post-BAS. BAS: balloon atrial septostomy; ECMO: extracorporeal membrane oxygenation; PaO_2_/FiO_2_: partial pressure of oxygen in arterial blood to the fraction of inspiratory oxygen concentration; VA-ECMO: veno-arterial extracorporeal membrane oxygenation.

All endpoints are listed in Table 2. Nine patients (82%) had an improvement in the PaO_2_/FiO_2_ ratios 24 h post-BAS procedure, while patients #8 and #9 had lower PaO_2_/FiO_2_ ratios due to concomitant pneumonia. Patient #11 demonstrated relatively similar pre- and post-procedural PaO_2_/FiO_2_ ratio (Figure 1). According to the official interpretation by a blinded radiologist, all patients demonstrated improvement in pulmonary edema on chest X-rays within 24 to 48 h post-procedure. Full resolution of pulmonary edema was observed in nine patients, with a mean resolution time of 7 days (range: 1.5 to 13 days). Patients #9 and #11 underwent continuous veno-venous hemofiltration (CVVH) 2 and 4 days before the BAS procedure, respectively. Patient #10 received CVVH the same day as the BAS, while patients #2 and #4 underwent CVVH 3 and 1 day after the septostomy, respectively. The ECMO flow improved in eight patients after the procedure, remained unchanged in patient #11, and decreased in patients #2 and #8.

**Figure 2:**
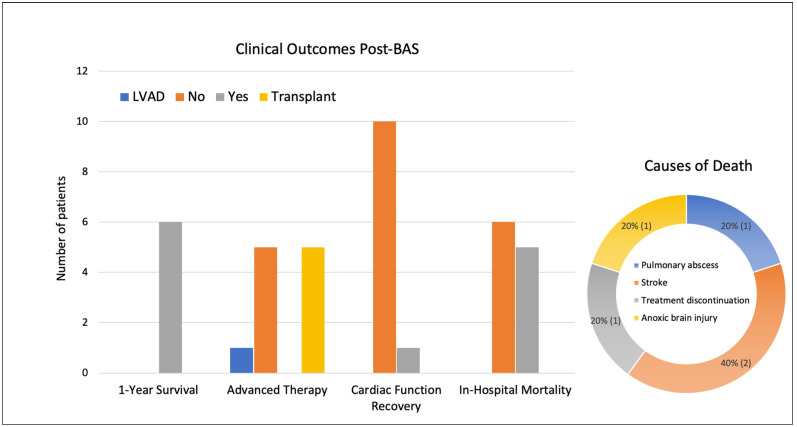
Clinical outcomes post-balloon atrial septostomy (BAS) and causes of death. The bar chart on the left shows the distribution of the clinical outcomes for patients who underwent a BAS while on VA-ECMO. The donut chart on the right shows the causes of mortality. BAS: balloon atrial septostomy; LVAD: left-ventricular assist device; VA-ECMO: veno-arterial extracorporeal membrane oxygenation.

The mean in-hospital and ICU length of stay were 32 days and 21 days, respectively. Five patients (45%) received heart transplants, with a mean time of 6.4 days following the BAS. One patient received LVAD 5 days after the BAS and one patient had cardiac function recovery, with the latter being the youngest patient (35 years old) with a diagnosis of myocarditis. Five patients (45%) died in the hospital, with a mean time to mortality of 11 days following the BAS. Two patients died of a massive stroke, one of an anoxic brain injury, and one had care discontinued due to ECMO weaning failure. Patient #11 was transferred to another center for an LVAD but died of a pulmonary abscess before the procedure. All six patients who survived to discharge were alive at the 1-year follow-up (Figure 2). Table 3 summarizes the key characteristics and outcomes for each patient who underwent a BAS.

## 4. Discussion

In this article, we present the procedural and clinical outcomes of 11 patients with cardiogenic shock on VA-ECMO who underwent a percutaneous BAS. Our major findings were as follows: (1) the BAS in adult patients on VA-ECMO was safe and effective, with a 100% procedural success rate and no procedural complications; (2) the BAS significantly improved pulmonary edema and oxygenation in the majority of patients, highlighting its utility as an LV unloading strategy (Graphical Abstract).

BAS was first introduced in 1966 by Rashkind et al. [6] for the palliative treatment of transposition of the great arteries. Over time, BAS became widely used for managing complex congenital heart diseases. In 1983, Rich et al. [7] applied BAS procedures to patients with refractory pulmonary hypertension and right heart failure. BAS has since proven effective in severe pulmonary arterial hypertension cases and is used to relieve left heart tension during VA-ECMO, thereby improving cardiac function [8]. Nonetheless, its use in the adult population with cardiogenic shock remains inadequately studied. Our experience, along with other studies (Table S1), confirms the feasibility and the safety of the percutaneous BAS. A successful BAS was achieved in 100% of patients with no complications. This technique does not leave any material in the body and does not expose the patient to an increased risk of infection, thrombosis, or complications associated with an indwelling device.

The literature on the optimal balloon size for BAS remains limited. Some studies have suggested that a larger ASD (24–28 mm) is associated with a more rapid resolution of LA hypertension and increased procedural success rates. However, in the largest study that evaluated BASs with a median balloon size of 28 mm, complications occurred in 9.4% of patients, where two cases had atrial perforations [8]. Other authors suggested that a smaller ASD is as effective, does not require closure or repair, and is better tolerated hemodynamically [9]. In our study, the balloon size selection was based on individual patient anatomy and interatrial septum dimensions. This individualized approach proved to be a safe strategy, minimizing the risk of septal tearing while ensuring adequate left atrial decompression.

A BAS is a powerful LV unloading tool. Previous case reports and small-size studies showed that a percutaneous BAS is both feasible and effective, predominantly in pediatric patients on VA-ECMO [10,11]. However, the impact of a BAS on LV unloading and overall cardiac function in adult patients with cardiogenic shock remains poorly understood. In our study, the majority of the cases showed resolution of the pulmonary edema, improved PaO_2_/FiO_2_ ratios, and ECMO flow improvement, highlighting that a BAS is effective for LV unloading, improving oxygenation, and reducing pulmonary congestion. In a porcine model under near maximal VA-ECMO support, atrial septostomy resulted in a rapid and significant reduction in LV work by 22%, primarily by decreasing the end-diastolic pressure, stroke volume, and end-systolic pressure [12]. These hemodynamic effects translate clinically into an improvement of pulmonary congestion that otherwise was refractory to medical treatment. All patients in our study exhibited signs of pulmonary edema improvement on chest X-ray 24–48 h after the BAS procedure. However, three patients had CVVH started the same day, 1 day, and 3 days after the procedure to help manage volume overload, which is a confounding factor for results interpretation. Two other patients had CVVH started 2 and 4 days before the BAS, but clinical improvement was only seen after the procedure. In our literature review, all studies that examined the impact of the BAS on patients’ chest X-rays reported either improvement or complete resolution of the pulmonary edema (Table S1). In our cohort, the PaO_2_/FiO_2_ ratio increased in most patients 24 h after the procedure, which confirmed that the septostomies improved oxygenation. Although the PaO_2_/FiO_2_ ratio and chest X-rays alone cannot distinguish causes of hypoxemia, rapid improvement (24–48 h) of these parameters supports the initial diagnosis of pulmonary edema. In fact, two of our patients had a decrease in the PaO_2_/FiO_2_ ratio because they had concomitant pneumonia. While our study focused solely on the outcomes of BASs for LA decompression in VA-ECMO-supported patients, future studies could explore comparisons between a BAS and other LV-venting strategies, such as Impella or surgical left-ventricular venting. Comparative analyses may provide insights into the relative effectiveness, safety, and long-term outcomes of these approaches, potentially guiding clinicians in selecting the most appropriate decompression strategy for patients with cardiogenic shock. Additionally, evaluating the cost-effectiveness and procedural risks associated with each method would further support evidence-based decision-making in this high-risk population.

The ECMO flow increased 24 h after the procedure in most patients, which may reflect a decrease in LV wall stress and, as a consequence, a decrease in the preload. The mortality rate in our study was 45%, and in other case series, ranged between 29–56% (Table S1). Mortality timing following the BAS was between 5 and 23 days, with none directly associated with the procedure. Most deaths were attributed to complications from the cardiogenic shock or the ECMO, highlighting the poor prognosis of these patients.

A septostomy reduces the LV workload and stroke volume, which may lead to a non-functioning LV, potentially causing blood stasis within the ventricular cavity or aortic root. This increases the risk of thrombus formation resulting in serious consequences. In our study, two patients died in the hospital due to a stroke. Thus, it is crucial to maintain adequate anticoagulation, carefully balancing the risks of bleeding versus thrombosis, while closely monitoring the hemodynamic status to prevent such adverse events.

In managing cardiogenic shock secondary to heart failure, the choice of mechanical circulatory support (MCS) device plays a pivotal role in optimizing outcomes and tailoring support to patient-specific needs. A recent review by Vlachakis et al. [13] underscored the distinct roles of MCS devices, such as the Intra-Aortic Balloon Pump, Impella, TandemHeart (TH; LivaNova Inc., Pittsburgh, PA, USA), and VA-ECMO, each offering different hemodynamic benefits and operational advantages. For instance, VA-ECMO provides comprehensive circulatory support but may increase LV afterload, thereby risking pulmonary edema and necessitating adjunctive LV unloading strategies. While direct LV unloading devices like the Impella effectively address left-sided overload by reducing LV pressure, devices such as TandemHeart, despite their effectiveness, require complex insertion techniques that limit their use to specialized centers.

In this landscape, BAS emerges as a targeted, minimally invasive alternative for LA decompression, particularly in patients on VA-ECMO, where it can alleviate LA pressure without additional LV mechanical intervention. The role of BAS as an adjunctive decompression option is significant for clinical decision-making, providing a flexible, low-risk alternative in cases where additional support is essential to managing LV overload. Our findings align with the literature in showing BAS’s feasibility and effectiveness in improving oxygenation and pulmonary status by enhancing left atrial venting [8,9]. Given the challenges associated with direct LV decompression in VA-ECMO patients, a BAS provides an accessible, less invasive option for unloading in high-risk individuals with severe LV overload. We recognize that while BAS does not replace more invasive unloading devices, it offers a complementary option that may benefit select patients in a multi-device MCS strategy. Table 4 outlines the specific protocols to consider for follow-up monitoring post-BAS to manage potential complications. Future research comparing the outcomes of BASs with other LV decompression techniques could help refine its role within the MCS framework and contribute to a more personalized approach to managing complex cardiogenic shock cases.

Our cohort included patients with diverse etiologies of cardiogenic shock, such as ischemic cardiomyopathy, myocarditis, and postpartum cardiomyopathy. These underlying causes may influence both the hemodynamic response and overall outcomes of BASs. While our sample size limited the feasibility of conducting a statistically robust subgroup analysis, this diversity highlights an important area for future research. Studies with larger populations could investigate differential outcomes based on cardiogenic shock etiology, potentially uncovering which patient groups benefit the most from BAS. Such findings could enable clinicians to tailor BAS use to specific patient profiles, thereby optimizing outcomes in this critically ill population.

## 5. Limitations

A primary limitation of this study was the absence of a comparison group of VA-ECMO patients who did not undergo BAS, which restricted our ability to evaluate the relative efficacy of BASs comprehensively. The small sample size of this study (11 patients) presented inherent statistical limitations, including reduced power to detect smaller effects and limited capacity for subgroup analyses. This restricted the generalizability of our findings and could have introduced greater variability in the outcomes. Detailed invasive hemodynamic data were not available for all patients. Also, while the main cause of cardiogenic shock in our study was acute coronary syndrome, other etiologies (e.g., myocarditis, and postpartum cardiomyopathy) were included. This heterogenicity may limit the interpretation of the results, as each etiology can evolve differently with variable responses to LV unloading.

## 6. Conclusions

This study demonstrated BAS as a feasible and effective strategy for LA decompression in adult VA-ECMO patients with cardiogenic shock, which resulted in a clinical improvement of pulmonary edema and oxygenation. However, further research through multicenter prospective studies is essential to validate these outcomes across broader populations. Future research should also aim to evaluate the long-term impacts, focusing particularly on LV function recovery and quality of life, to fully elucidate BAS’s role in the management of this critically ill patient group.

## Figures and Tables

**Table 1:** Baseline and procedural characteristics.

Patient #	1	2	3	4	5	6	7	8	9	10	11
Age, years	50	50	55	58	61	68	60	35	63	40	56
Sex	M	M	F	M	M	M	M	F	M	F	M
BSA, m^2^	1.99	2.24	1.62	2.18	1.82	1.83	1.02	1.61	1.94	1.85	2.12
Arterial Hypertension	No	Yes	No	No	Yes	Yes	No	No	Yes	No	No
Diabetes	Yes	No	No	Yes	Yes	No	No	No	No	No	No
Dyslipidaemia	No	Yes	No	No	Yes	No	No	No	No	No	No
Chronic kidney disease	No	No	No	No	No	No	No	No	No	No	No
Prior myocardial infarction	No	No	No	No	Yes	No	No	No	No	No	No
Prior PCI	No	No	No	No	No	Yes	No	No	No	No	No
Prior CABG	No	No	No	No	No	No	No	No	No	No	No
HFrEF	No	No	No	No	Yes	Yes	No	No	No	No	No
Prior hospitalization for HF	No	No	No	No	No	No	No	No	No	No	No
Prior stroke or TIA	No	No	No	No	No	No	No	No	No	No	No
Peripheral arterial disease	No	No	No	No	No	No	No	No	No	No	No
Cause of cardiogenic shock	Ischemia	Ischemia	Ischemia	Ischemia	Ischemia + arrhythmia	Ischemia	Ischemia	Myocarditis	Arrhythmia	Postpartum	Ischemic transplant dysfunction
Cardiac arrest before ECMO	Yes	Yes	Yes	No	No	No	No	No	No	Yes	No
Pre-ECMO LVEF	15	20	0	15	15	5	10	5	5	5	NA
Pre-ECMO cardiac device support	IABP	No	IABP	No	No	IABP	No	No	No	No	No
ECMO duration, days	17	8	2	11	5	8	6	7	12	24	NA
Pre-BAS CVVH, days	-	-	-	-	-	-	-	-	2	-	4
Post-BAS CVVH, days	-	3	-	1	-	-	-	-	-	0	-
Procedural Characteristics											
Time from ECMO to BAS, days	3	6	0	4	0	4	1	2	2	1	1
Balloon diameter	Powerflex 12 mm	Boston scientific esophageal 18 mm	Powerflex 12 mm	Balloon z-med 20 mm	Powerflex pro 8 mm	Balloon z-med II 24 mm	Balloon Medtronic 12 mm	Balloon z-med II 20 mm	Balloon z-med II 16 mm	Boston Scientific OTW charger 12 mm	Balloon powerflex pro 12 mm
Size of ASD on TEE, mm	10	18	12	15	8	14	11	10	16	11	12
Procedural success	Yes	Yes	Yes	Yes	Yes	Yes	Yes	Yes	Yes	Yes	Yes
Procedure time, min	50	36	50	65	30	34	40	70	57	66	60
Procedural complications	No	No	No	No	No	No	No	No	No	No	No

ASD: atrial septal defect; BAS: balloon atrial septostomy; BSA: body surface area; CABG: coronary artery bypass grafting; CVVH: continuous veno-venous hemofiltration; ECMO: extracorporeal membrane oxygenation; HF: heart failure; HFrEF: heart failure with reduced ejection fraction; IABP: intra-aortic balloon pump; LVEF: left ventricular ejection fraction; PCI: percutaneous coronary intervention; TIA: transient ischemic attack; TEE: transesophageal echocardiography.

**Table 2:** Patient outcomes.

Patient #	1	2	3	4	5	6	7	8	9	10	11
LA pressure, mmHg											
Pre-BAS	NA	47/49 (56)	NA	NA	NA	NA	NA	NA	NA	NA	43/57 (36)
Post-BAS	15/11 (10)	26/22 (17)	NA	NA	NA	NA	NA	NA	NA	NA	34/40 (32)
LVEDD, mm											
Pre-BAS	58	50	NA	58	59	52	72	41	64	77	NA
Post-BAS	NA	NA	NA	NA	52	NA	NA	48	57	79	NA
PaO_2_/FiO_2_											
Pre-BAS	195	289	462	242	81	372	171	617	262	220	365
24 h post-BAS	212	298	797	430	363	483	254	300	101	238	348
Clear CXR, days	1	6	9	13	4	4	Improved but not resolved	9	10	Improved but not resolved	3
ECMO flow, L/min											
Pre-BAS	4.07	4.8	2.81	2.81	4.03	4.8	3.46	3.44	3.58	3.62	4.27
24 h post-BAS	4.13	4.36	3.46	3.79	4.26	5.02	3.57	3.1	3.83	3.87	4.29
Cardiac function recovery (LVEF > 40%)	No	No	No	No	No	No	No	Yes	No	No	No
Advanced therapy	Transplant	Transplant	Transplant	Transplant	LVAD	Transplant	No	No	No	No	No
Time to advanced therapy (from BAS), days	14	2	2	10	5	4	-	-	-	-	-
In-hospital mortality	No	No	No	No	No	Yes	Yes	No	Yes	Yes	Yes
Cause of death	-	-	-	-	-	Massive brainstem ischemic stroke after transplant	Massive hemorrhagic stroke	-	Treatment discontinuation due to ECMO weaning failure	Anoxic brain injury	Pulmonary abscess
Time to mortality (from BAS), days	-	-	-	-	-	6	5	-	10	23	NA
In-hospital length of stay, days	70	69	14	46	58	9	6	17	12	23	NA
ICU length of stay, days	50	32	10	27	32	9	6	10	12	23	NA
30-day mortality	No	No	No	No	No	-	-	No	-	-	-
1-year survival	Yes	Yes	Yes	Yes	Yes	-	-	Yes	-	-	-

BAS: balloon atrial septostomy; ECMO: extracorporeal membrane oxygenation; CXR: chest X-ray; ICU: intensive care unit; LA: left atrium; LVAD: left-ventricular assist device; LVEF: left-ventricular ejection fraction; NA: not available; PaO_2_/FiO_2_: partial pressure of oxygen in arterial blood to the fraction of inspiratory oxygen concentration.

**Table 3:** Summary of patient characteristics and outcomes.

Patient #	Age	Sex	Etiology of Shock	Balloon Size (mm)	Procedural Success	PaO_2_/FiO_2_ Improvement	Pulmonary Edema Resolution (Days)	In-Hospital Outcome	1-Year Follow-Up
1	50	M	Ischemic cardiomyopathy	12	Yes	Yes	1	Discharged	Survived
2	50	M	Ischemic cardiomyopathy	18	Yes	Yes	6	Discharged	Survived
3	55	F	Ischemic cardiomyopathy	12	Yes	Yes	9	Discharged	Survived
4	58	M	Ischemic cardiomyopathy	20	Yes	Yes	13	Discharged	Survived
5	61	M	Ischemic + arrhythmogenic cardiomyopathy	8	Yes	Yes	4	Discharged	Survived
6	68	M	Ischemic cardiomyopathy	24	Yes	Yes	4	Mortality	-
7	60	M	Ischemic cardiomyopathy	12	Yes	Yes	-	Mortality	-
8	35	F	Myocarditis	20	Yes	No	9	Discharged	Survived
9	63	M	Arrhythmogenic cardiomyopathy	16	Yes	No	10	Mortality	-
10	40	F	Postpartum cardiomyopathy	12	Yes	Yes	-	Mortality	-
11	56	M	Ischemic cardiomyopathy	12	Yes	No	3	Mortality	-

PaO_2_/FiO_2_: partial pressure of oxygen in arterial blood to the fraction of inspiratory oxygen concentration.

**Table 4:** Follow-up protocol for patients post-BAS.

Time	Post-BAS	Assessment Details
24–48 h	Oxygenation status and imaging	Monitor PaO_2_/FiO_2_ ratios and perform chest X-rays to assess the resolution of pulmonary edema.
	Hemodynamic monitoring	Continuous monitoring of LV pressure, where feasible, with echocardiography to assess LV decompression and ensure stable ECMO flows.
1 week	Echocardiography	Re-assess LV and RV function, left atrial size, and pulmonary congestion resolution to detect any signs of ventricular overload early.
	Blood chemistry	Evaluate renal function, serum lactate, hemoglobin, and electrolytes for signs of systemic perfusion adequacy and metabolic stability.
1 month	Echocardiography	Monitor changes in LV dimensions, contractility, and LA volume. Detect early signs of LV hypertrophy or structural changes.
	Clinical evaluation	Physical assessments for signs of HF recurrence or fluid retention; review symptomatology improvements, especially respiratory function.
3–6 months	Advanced imaging (e.g., MRI)	Detailed imaging to track structural or functional changes in LV and LA, including wall thickness and fibrosis.
	Quality of life and functional status	Monitor improvement in exercise tolerance and overall quality of life. Questionnaire-based assessments (NYHA class, 6 min walk test).

BAS: balloon atrial septostomy; ECMO: extracorporeal membrane oxygenation; HF: heart failure; LA: left atrium; LV: left ventricle; MRI: magnetic resonance imaging; NYHA: New York Heart Association; PaO_2_/FiO_2_: partial pressure of oxygen in arterial blood to the fraction of inspiratory oxygen concentration; RV: right ventricle.

## Data Availability

All data supporting this article will be shared upon reasonable request to the corresponding author.

## Supplementary Materials

The following supporting information can be downloaded from https://www.mdpi.com/article/10.3390/jcm13237433/s1: Table S1. Literature summary of BAS in patients on VA-ECMO [9,10,14,15,16,17,18,19].

## Abbreviations

BAS	Balloon atrial septostomy
CVVH	Continuous veno-venous hemofiltration
ECMO	Extracorporeal membrane oxygenation
LA	Left atrium
LV	Left ventricle/ventricular
LVAD	Left-ventricular assist device
VA	Venoarterial
