# Supplementary material for: Percutaneous Atrial Septostomy in Adult Patients on Veno-Arterial Extracorporeal Membrane Oxygenation for Cardiogenic Shock: A Canadian Single-Center Experience
Source: J Clin Med. 2024 Dec 6;13(23):7433. doi: 10.3390/jcm13237433 (PMC11642157; doi:10.3390/jcm13237433)
Supplement: Supplementary file 1 [file jcm-13-07433-s001.zip › jcm-3286264-supplementary.pdf]

Supplementary Appendix

1. Supplementary tables

1.1 Table S1………………………………………………………….. p. 2

1.2 References……………………………………………………….. p. 4

Table S1. Literature summary of BAS in patients with VA-ECMO

Author	Years	Study design	Procedure	Sample size, no.	Mean age (years)	Mean LVEF %	Days from ECMO to septostomy	Procedural Success (%)	Outcomes	In hospital mortality (%)	Function recovery versus advanced therapy (n)	Comments
LinYN et al. [1]	2012 - 2014	Retro case series	Percutaneous atrial septostomy By Inoue Balloon 24-27 mm	15	48.3	15	4.3	93	1) rapid resolution of pulmonary oedema on CXR 2) reduction of oxygenation index	46.6	Heart transplant: 4 Function recovery: 4 LVAD: 1	-6 patients died of sepsis. -1 died of ischemic bowel disease
Alhussein M et al. [2]	2010 - 2016	Retro case series	Percutaneous atrial septostomy By Mustang Balloon 20 mm	7	33	<20	1.3	100	Improvement of pulmonary oedema on CXR	29	Heart transplant: 2 Function recovery: 1 LVAD: 2	-1 patient died of MOF -1 died after withdrawal of care for severe anoxic brain injury
Amancherla K et al. [3]	2018 - 2020	Retro case series	Percutaneous balloon atrial septostomy 12-15 mm	12	48	13.8	NA	100	1) reduction of mean LA pressure 2) improvement of pulmonary oedema in most patients 3) improvement of ECMO flow	42	heart transplant: 4 function recovery: NA LVAD: 1	-Cause of death not mentioned - Smaller ASD defect (15mm) is better tolerated, with fewer complications; and no ASD closure is needed
Prasad A et al. [4]	2011 - 2016	Retro case series	Percutaneous balloon atrial septostomy	9	46	NA	NA	100	1) decrease of LA pressure 2) increase of PaO2/FiO2 3) Improvement of pulmonary oedema on CXR	56	Function recovery: 2 LVAD:2	5 patients had withdrawal of care (2 for severe MOF, 2 for medical futility, 1 declined LVAD)
Dahdouh Z et al. [5]	2012	Case report	Percutaneous balloon atrial septostomy	1	49	10	NA	100	Improvement of pulmonary oedema and decrease of invasive central venous pressure	0	Heart transplant	-Cardiogenic shock due to acute myocardial infarction. -Successful heart transplant performed later
Dahdouh Z et al. [6]	2012	Case report	Percutaneous balloon	1	47	15	NA	100	1) improvement of pulmonary edema	0	Complete function recovery	Cardiogenic shock due to

			atrial septostomy						2) decrease in mean LA pressure<br>3) better ECMO output			H1N1 infection
Katamreddy A et al. [7]	-	Case report	Percutaneous atrial septostomy	1	62	-	-	100	Clinical improvement	0	Full recovery	
Gültekin S et al. [8]	-	Case report		1	49	-	10	100	Decrease of mean pulmonary pressure	0	Heart transplant	Case series of 2 patients, but only one had BAS

ASD: atrial septal defect; BAS: balloon atrial septostomy; ECMO: extracorporeal membrane oxygenation; CXR: chest X-ray; ICU: intensive care unit; LA: left atrium; LVAD: left ventricular assist device; LVEF: left ventricular ejection fraction; MOF: multiple organ failure; NA: not available; PaO_2/FiO_2: partial pressure of oxygen in arterial blood to the fraction of inspiratory oxygen concentration

Supplementary references

1. Lin YN, Chen YH, Wang HJ, Hung JS, Chang KC, Lo PH. Atrial Septostomy for Left Atrial Decompression During Extracorporeal Membrane Oxygenation by Inoue Balloon Catheter. Circ J. 2017 Sep 25;81(10):1419-1423. doi: 10.1253/circj.CJ-16-1308. Epub 2017 May 11. PMID: 28496031.
2. Alhussein M, Osten M, Horlick E, Ross H, Fan E, Rao V, Billia F. Percutaneous left atrial decompression in adults with refractory cardiogenic shock supported with veno-arterial extracorporeal membrane oxygenation. J Card Surg. 2017 Jun;32(6):396-401. doi: 10.1111/jocs.13146. Epub 2017 May 11. PMID: 28497496.
3. Amancherla K, Menachem JN, Shah AS, Lindenfeld J, O'leary J. Limited Balloon Atrial Septostomy for Left Ventricular Unloading in Peripheral Extracorporeal Membrane Oxygenation. J Card Fail. 2021 Apr;27(4):501-504. doi: 10.1016/j.cardfail.2020.12.014. Epub 2021 Jan 16. PMID: 33358956.
4. Prasad A, Ghodsizad A, Brehm C, Kozak M, Körner M, El Banayosy A, Singbartl K. Refractory Pulmonary Edema and Upper Body Hypoxemia During Veno-Arterial Extracorporeal Membrane Oxygenation-A Case for Atrial Septostomy. Artif Organs. 2018 Jun;42(6):664-669. doi: 10.1111/aor.13082. Epub 2018 Jan 18. PMID: 29344963.
5. Dahdouh Z, Roule V, Sabatier R, Lognoné T, Labombarda F, Pellissier A, Belin A, Ivascau C, Buklas D, Massetti M, Grollier G. Extra-corporeal life support, transradial thrombus aspiration and stenting, percutaneous blade and balloon atrioseptostomy, all as a bridge to heart transplantation to save one life. Cardiovasc Revasc Med. 2012 Jul-Aug;13(4):241-5. doi: 10.1016/j.carrev.2012.02.007. Epub 2012 Apr 3. PMID: 22480784.
6. Dahdouh Z, Roule V, Lognone T, Sabatier R, Massetti M, Grollier G. Atrial septostomy in cardiogenic shock related to H1N1 infection. Acute Card Care. 2013 Mar;15(1):7-9. doi: 10.3109/17482941.2012.760740. PMID: 23425007.
7. Katamreddy A, Snipelisky DF, Eleid MF. Atrial Septostomy as a Bridge to Replace a Thrombosed Mechanical Aortic Valve Requiring Extracorporeal Membrane Oxygenation. J Heart Valve Dis. 2016 Sep;25(5):644-647. PMID: 28238250.
8. Gültekin B, Ersoy Ö, Akkaya İ, Kayıpmaz Ç, Pirat A, Sezgin A. Decompression of Left Ventricle During Venoarterial Extracorporeal Membrane Oxygenation Support as a Step to Transplant. Exp Clin Transplant. 2016 Nov;14(Suppl 3):42-44. PMID: 27805509.
